# A Global Interconnected Observer for Attitude and Gyro Bias Estimation with Vector Measurements

**DOI:** 10.3390/s20226514

**Published:** 2020-11-14

**Authors:** Huijuan Guo, Huiying Liu, Xiaoxiang Hu, Yan Zhou

**Affiliations:** School of Automation, Northwestern Polytechnical University, Xi’an 710072, China; lhy2005@nwpu.edu.cn (H.L.); xxhu@nwpu.edu.cn (X.H.); lea@mail.nwpu.edu.cn (Y.Z.)

**Keywords:** attitude estimation, nonlinear observer, global exponential stability, robustness, navigation, low-cost sensor

## Abstract

This paper proposes a novel interconnected observer to get good estimates of attitude and gyro bias from high-noise vector measurements. The observer is derived based on the theory of nonlinear and linear cascade systems, and its error dynamics have the properties of global exponential stability and robustness to bounded noise. These properties ensure the convergence and boundedness of the attitude and gyro bias estimation errors. To obtain higher estimation accuracy, an approach to calculate time-varying gains for the proposed auxiliary observer is designed under the premise of considering noise terms in the rate gyro and vector sensors. The simulation results show that when the vector sensors’ outputs contain high-level noise, the proposed observer with time-varying gains yields better performance in both the transient and steady-state phases.

## 1. Introduction

Attitude estimation of a rigid body is an indispensable part of navigation. Questions of estimating attitude have been a field of concern for decades due to its numerous applications in various systems, such as unmanned underwater vehicles (UUVs) [1], unmanned aerial vehicles (UAVs) [2], and others [3]. A rigid body’s attitude can be resolved by integrating the angular velocity from a rate gyro output. However, even with high-precision gyros, the accumulated drift over time can affect the accuracy of the attitude estimation, not to mention the low-cost ones. A typical approach to estimate attitude is to utilize algebraic methods of vector measurements only by comparing vectors measured in either the body-fixed coordinate frame or the reference frame with vectors measured in the other. Triad and Quest in [4] used two or more nonparallel vector measurements to determine the attitude. Unfortunately, bias and noise can easily corrupt the vector measurements. Therefore, combining angular velocity sensors with vector sensors (e.g., accelerometers, magnetometers, star trackers, or sun sensors) has been developed for improving the estimation accuracy.

The current approaches for estimating attitude and gyro bias from vector sensors and rate gyros can be summarized into two classes, stochastic filtering algorithms (such as EKF, UKF, and their variants; see [5,6,7]) and nonlinear observers (e.g., [8,9]). Although EKFs and UKFs have been widely used, they cannot guarantee convergence in strongly nonlinear systems, and UKFs may increase the computational cost. In recent years, more efforts have been made in constructing nonlinear observers. In [10,11], nonlinear complementary filters designed on the special orthogonal group SO(3) for low-cost measurement units became the foundation of other observers. On this basis, Grip et al. [12] developed semi-globally stable observers with gyro bias and time-varying reference vectors. After that, they came up with an alternative semi-global attitude observer in the unit quaternion space [13] by employing the theory of cascaded linear and nonlinear system observers, which appeared in [14,15]. For obtaining globally stable observers, some studies adopt the SO(3) topological constraint lifting methods before estimating the attitude rotation matrix, such as Batista et al. [16,17], Grip et al. [9,18], Bryne et al. [19], and Fusini et al. [20]; in addition, the nonlinear observers in [18,19,20] are all based on extensions or various applications of the observer in [9]. Among these observers, except the observer in [16] attained global asymptotical stability; others achieved global exponential stability. Besides et al. [21,22] proposed two different “geometry-free” nonlinear observers and verified that the former has the property of global asymptotic stability, while the latter has global exponential stability. All the aforementioned nonlinear observers apply at least two vector measurements combined with gyro measurements. Unlike them, the methods described by Batista et al. [23,24] merely use a signal vector observation, coupled with a persistency-of-excitation (PE) condition, and also reach global exponential stability.

Most observers mentioned the preceding are designed and proved under the assumption of noise-free scenarios and do not allow vector measurement bias. Like [25,26,27], researchers took noise terms of the linear cascade system into account just when deriving and calculating the time-varying gains of the linear system observer, which means they do not consider the gyro and vector sensor noise. Therefore, a noisy attitude observer model was given in [28] while discussing and proofing the cascade attitude observer’s robustness to bounded noise and stochastic noise on all sensors. Additionally, [12] first offered a method of estimating body-fixed vector bias, using an exponential convergence algorithm, and then employing this method in cascade attitude and gyro bias observers to improve the estimation accuracy. Moreover, Martin et al. [29] presented a nonlinear “non-geometry” observer, regarded a bias of one vector observation and rate gyro. They verified that considering one vector measurement bias can also obtain global asymptotic stability and local exponential stability. The literature we listed has estimated the gyro bias on the premises that the rate gyro exists, and its accumulated drift is controllable. If those premises are not true, another trend requiring attention is angular velocity reconstruction without applying rate gyros to obtain reasonable gyro bias estimation. Recently, relatively simple angular velocity estimation observers have been proposed by Magnis and Petit [30,31]. These observers reconstruct the angular velocity directly from the vector measurements, even a single vector measurement, without any attitude information or gyro measurements, and quickly convergence. However, users need to assume that all the torques applied to the rigid body are known. To solve this problem, Magnis et al. [32] extended their work to estimate torques even with varying rotation rates and unknown direction. Notably, these angular velocity estimation observers can make a positive contribution to gyro bias estimation.

We consider the attitude and gyro bias estimation using a rate gyro and two or more vector sensors. Built on the theory of Grip et al. [15] for cascaded nonlinear and linear systems, the primary contribution of this paper is to construct an interconnected nonlinear observer to estimate attitude and gyro bias from the re-estimated vector measurements. The proposed observer includes two cascaded subsystems: a nonlinear attitude and gyro bias observer modified from the nonlinear observer in [9], and a linear vector measurement observer devised to filter the vector measurements, called an auxiliary observer. This paper analyzes the proposed observer’s stability and gives its noisy error dynamics to study its robustness. When proving its robustness to bounded noise, the input-to-state stability theorem is used, and all the sensors’ noise is regarded. Beyond that, the time-varying gains computed from the discrete Riccati equation, for the auxiliary observer, is designed to improve its accuracy and robustness. This model also thinks about noise on all sensors.

We begin with the required sensor models and assumptions in Section 2. We design the globally interconnected observer and analyze its stability in Section 3. Section 4 briefly proves the proposed observer’s robustness to bounded noise. Section 5 discusses the results of the numerical simulation with several cases in detail. Section 6 concludes this paper.

### Notation and Preliminaries

The operator ${\left(\cdot \right)}^{T}$ denotes the transpose of a vector or matrix; ‖⋅‖ represents the vectors’ Euclidean norm and the matrices’ Frobenius norm; and E[⋅] forms the expectation of its inputs. The symbols $I_{n\times n}$ and $0_{m\times n}$ denote the n×n identity matrix and the m×n zero element matrix, respectively. For a vector ${\mathbb{R}}^{3}$, defined as $r={\left[\begin{array}{ccc} r_{1} & r_{2} & r_{3} \end{array}\right]}^{T}$, its skew-symmetric is denoted by S(r), where

(1) $$S\left(r\right)=\left[\begin{array}{ccc} 0 & -r_{3} & r_{2}\\ r_{3} & 0 & -r_{1}\\ -r_{2} & r_{1} & 0 \end{array}\right],$$

so that for any $a\in {\mathbb{R}}^{3}$, S(r)a=r×a, where × means the vector cross product. Given a linear function, vex(⋅) denotes the inverse operation of S(r), such that vex(S(r))=r. For a square matrix U, ${\mathbb{P}}_{a}\left(U\right)=\left(U-U^{T}\right)/2$ denotes its skew-symmetric part, tr(U) means its trace, $\left|\mathrm{tr}\left(U\right)\right|\leq \sqrt{3}\left\|U\right\|$; for a symmetric matrix U, tr(US(r))=0. Furthermore, the trace of a skew-symmetric matrix is zero, and there exists $\mathrm{tr}\left(S^{T}\left(r\right)S\left(a\right)\right)=2r^{T}a$. A block-diagonal matrix $U_{3}$ of two square matrices $U_{1}$ and $U_{2}$ is indicated with $U_{3}=\mathrm{blkdiag}\left(U_{1},U_{2}\right)$. The saturation operation of a vector or matrix argument to the interval [−L,L] is performed by ${\mathrm{sat}}_{L}\left(\cdot \right)$. Proj(⋅) is a projection operator whose function here is to preserve its inputs in a predefined bound.

In general, we need two coordinate frames to describe the attitude: the body-fixed frame and the reference frame. The body-fixed frame, denoted by {B}, is on the rigid body; its origin is rigorously fastened to the body’s center of mass. The reference frame is ordinarily unmoving, called an inertial frame {I}. Here, the north–east–down (NED) navigation frame, denoted by {N}, is used as the inertial frame, which means that the rotation of the Earth would be ignored in low-precision applications. $R_{B}^{N}\in SO\left(3\right)$ represents a rotation matrix from {B} to {N}, where SO(3) is defined by $\mathrm{SO}\left(3\right):=\left\{U\in {\mathbb{R}}^{3\times 3}|\det\left(U\right)=1,\;UU^{T}=U^{T}U=I_{3}\right\}$. For convenience, let $R\equiv R_{B}^{N}$. Furthermore, it is known that a vector norm remains unchanged after rotation, i.e., ‖Ra‖=‖a‖. By using superscript indexes to represent a vector decomposed in different coordinate frames, we have $x^{N}$ as the component of x in frame {N}, and $x^{B}$ is the same. According to the definition of R, the relationship between the above vector components can be written as $x^{N}=Rx^{B}$.

## 2. Problem Statement

### 2.1. Sensor Model

Consider a rigid body consisting of a triaxial rate gyro and two or more additional vector sensors, such as accelerometers and magnetometers. The attitude kinematics about the rotation matrix satisfy

(2) $$\dot{R}=RS\left(\omega \right),$$

where $\omega \in {\mathbb{R}}^{3}$ is the angular velocity of {B} with reference to {N}, expressed in {B}, and [33] gave its normal model:

(3) $$\omega =\omega_{m}-b_{\omega }-\eta_{\omega },$$

(4) $${\dot{b}}_{\omega }=0,$$

where $\omega_{m}$ is the outputs of the gyro; $b_{\omega }\in {\mathbb{R}}^{3}$ is a constant gyro bias vector; and $\eta_{\omega }$ is the sensor noise.

As described, at least two other sensors provide nonparallel vector measurements, so let M≥2, and suppose that two sets of M vector measurements $\left\{v_{i}\in {\mathbb{R}}^{3},i=1,\ldots ,M\right\}$ and $\left\{v_{mi}\in {\mathbb{R}}^{3},i=1,\ldots ,M\right\}$ in the body-fixed frame are available. The measurement model is

(5) $$v_{mi}=v_{i}+\eta_{i},$$

where $v_{mi}\in {\mathbb{R}}^{3},i=1,\ldots ,M$ denotes the corresponding sensor outputs; and $\eta_{i}\in {\mathbb{R}}^{3},i=1,\ldots ,M$ is the sensor noise on measurement i. The vector measurement $v_{i}$, in the body-fixed frame, of the known constant vectors $v_{i}^{N}$, when expressed in the reference frame (NED frame), conform to the following relationship:

(6) $$v_{i}=R^{T}v_{i}^{N}.$$

In this paper, vector measurements stand for a set of vectors measured in the body-fixed frame from other vector sensors, except for rate gyros, and satisfy Equation (6).

Omit the noisy terms in Equations (3)–(5) and substitute Equations (3) and (6) into Equations (2) and (5), respectively. We can get the noisy-free nonlinear system model as follows:

(7) $$\left\{ \begin{array}{l} \dot{R}=RS\left(\omega_{m}-b_{\omega }\right)\\ {\dot{b}}_{\omega }=0\\ v_{i}=R^{T}v_{i}^{N},\;i=1,\ldots ,M \end{array}\right.,$$

where $\omega_{m}=\omega +b_{\omega }$ in this noiseless system. The following observer design also utilizes this noiseless system.

### 2.2. Assumptions

Throughout this work, consider the following assumptions:

**Assumption** **1.** *At least two vectors in the body-fixed frame are not parallel to each other, i.e., there exists a constant γ>0, for all t≥0, there are i,j∈1,…,M and i≠j, such that $\left\|v_{i}\times v_{j}\right\|\geq \gamma >0$.*

**Assumption** **2.** *The gyro output signal $\omega_{m}$ and its derivative ${\dot{\omega }}_{m}$ are bounded for all t≥0.*

**Assumption** **3.** *The gyro bias $b_{\omega }$ is constant; there exists a known constant $l_{b}>0$, such that $\left\|b_{\omega }\right\|\leq l_{b}$.*

The first two assumptions are standard assumptions in attitude estimation; see, e.g., [12,13,17,20]. Assumption 1 is necessary to guarantee uniform observability. Assumption 3 is about gyro bias; also, we assume that $v_{i}^{N},\;i=1,\ldots ,M$ and ${\dot{v}}_{i}^{N},\;i=1,\ldots ,M$ are all continuous at t and uniformly bounded.

In this paper, we choose two non-collinear vector measurements denoted by $v_{1}$ and $v_{2}$ in the body-fixed frame, corresponding to $v_{1}^{N}$ and $v_{2}^{N}$ in the inertial frame, where $v_{1}^{N}$ and $v_{2}^{N}$ are also independent of each other; the above assumptions are equally applicable.

## 3. Nonlinear Interconnected Observer Design

**Figure 1:**
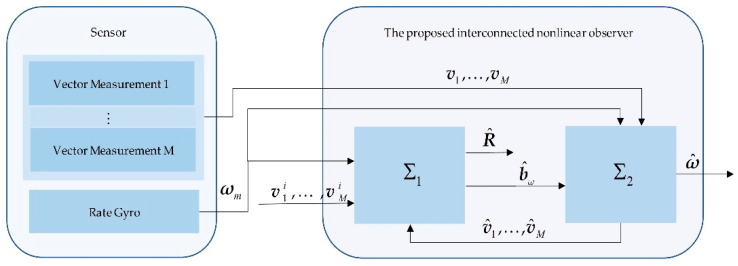
Overall structure of the total observer. $\sum_{1}$ denotes the attitude observer. $\sum_{2}$ denotes the auxiliary observer.

As shown in Figure 1, the proposed nonlinear interconnected observer includes two subsystems. The first subsystem is an attitude observer, which estimates the rotation matrix and gyro bias established on comparing at least two nonparallel vector measurements in two corresponding coordinate frames. If the attitude observer directly applies the vector sensors’ outputs as its input, the injection term will contain the vector measurement noise. This injection noise can change the accuracy of the attitude observer. Therefore, to reduce the impact of the measurement noise, we construct the second subsystem, an auxiliary observer for estimating the vector measurements and feeding the estimated value back to the attitude observer. The interconnected system $\sum_{1}-\sum_{2}$ is based on the theory of Grip in [15].

### 3.1. Subsystem $\sum_{1}$: Attitude Observer

The general framework of $\sum_{1}$ introduced here for the rotation matrix R and gyro bias $b_{\omega }$ is similar to that in Grip et al. [18], expressed as

(8) $$\sum_{1}:\left\{ \begin{array}{l} \dot{\hat{R}}=\hat{R}S\left(\omega_{m}-{\hat{b}}_{\omega }\right)+\theta K_{P}\Gamma \\ {\dot{\hat{b}}}_{\omega }=\mathrm{Proj}\left(\left\|{\hat{b}}_{\omega }\right\|\leq l_{\hat{b}},-k_{v}\mathrm{vex}\left({\mathbb{P}}_{a}\left({\hat{R}}_{s}^{T}K_{P}\Gamma \right)\right)\right) \end{array}\right.,$$

where θ, $K_{P}$, and $k_{v}$ are tunable parameters, tuned to obtain stability. In more detail, θ≥1 is a scaling factor; $K_{P}$ is a symmetric positive–definite gain matrix; and $k_{v}>0$ is a scalar gain. Function Proj(⋅) denotes a parameter projection operator to ensure the estimated gyro bias is bounded by $\left\|\hat{b}\right\|\leq l_{\hat{b}}$, see [9] for detail; and $l_{\hat{b}}$ is a constant, $l_{\hat{b}}>l_{b}$. ${\hat{R}}_{s}={\mathrm{sat}}_{1}\left(\hat{R}\right)$. Referencing the TRIAD algorithm [4], the injection term Γ is defined by

(9) $$\Gamma \left(v_{1},v_{1}^{N},v_{2},v_{2}^{N},\hat{R}\right):=A_{N}A_{B}^{T}-A_{N}A_{N}^{T}\hat{R},$$

(10) $$A_{B}:=\left[\begin{array}{ccc} v_{1} & v_{2} & v_{1}\times v_{2} \end{array}\right],$$

(11) $$A_{N}:=\left[\begin{array}{ccc} v_{1}^{N} & v_{2}^{N} & v_{1}^{N}\times v_{2}^{N} \end{array}\right],$$

which is different from the design strategy of $J:=A_{N}A_{B}^{T}-\hat{R}A_{B}A_{B}^{T}$ in [18]. Note that $A_{B}$ and $A_{N}$ are defined to fulfill Assumption 1. Besides, in the attitude observer, the rotation matrix $\hat{R}$ does not strictly adhere to the topological structure of SO(3).

**Property** **1.** *For all t>0, $A_{B}$ and $A_{N}$ are piecewise continuous in t and uniformly bounded by$\left\|A_{N}\right\|=\left\|A_{B}\right\|\leq L_{A}$. For all t>0, there is a constant $\beta_{1}>0$, such that $A_{N}A_{N}^{Τ}\geq \beta_{1}I_{3\times 3}$.*

Define the estimation errors of the rotation matrix and gyro bias as $\tilde{R}=R-\hat{R}$, ${\tilde{b}}_{\omega }=b_{\omega }-{\hat{b}}_{\omega }$. The error dynamics can be written as

(12) $$\left\{ \begin{array}{l} \dot{\tilde{R}}=RS\left(\omega_{m}-b_{\omega }\right)-\hat{R}S\left(\omega_{m}-{\hat{b}}_{\omega }\right)-\theta K_{P}\Gamma \\ {\dot{\tilde{b}}}_{\omega }=-\mathrm{Proj}\left({\hat{b}}_{\omega },-k_{v}\mathrm{vex}\left({\mathbb{P}}_{a}\left({\hat{R}}_{s}^{T}K_{P}\Gamma \right)\right)\right) \end{array}\right..$$

**Lemma** **1.** For any choice of $K_{P}>0$ and $k_{v}>0$, there exists a constant $\theta^{*}\geq 1$, such that, for all $\theta \geq \theta^{*}$, the origin $\tilde{R}=R-\hat{R}=0$ and ${\tilde{b}}_{\omega }=b_{\omega }-{\hat{b}}_{\omega }=0$ of Equation (12) is global exponential stability (GES).

**Proof.**  Having that $A_{B}=R^{T}A_{N}$, we can rewrite $\Gamma =A_{N}A_{N}^{T}\tilde{R}$, thus $\left\|\Gamma \right\|=\left\|A_{N}A_{N}^{T}\tilde{R}\right\|\leq {\left\|A_{N}\right\|}^{2}\left\|\tilde{R}\right\|\leq L_{A}^{2}\left\|\tilde{R}\right\|$. Hence, ‖Γ‖ in the present observer also remains linear in $\left\|\tilde{R}\right\|$, so the remaining proof is analogous to [18]. □

To reduce the vector measurement noise injection, we utilized the estimated vector measurements instead of the output signals of the vector sensors. Therefore we replaced every occurrence of $v_{1}$ and $v_{2}$ in $A_{B}$ by the estimated states ${\hat{v}}_{1}$ and ${\hat{v}}_{2}$, using ${\hat{A}}_{B}$ to replace $A_{B}$ in term Γ. Thus, $\hat{\Gamma }$ and ${\hat{A}}_{B}$ take the following forms:

(13) $$\hat{\Gamma }\left({\hat{v}}_{1},v_{1}^{N},{\hat{v}}_{2},v_{2}^{N},\hat{R}\right):=A_{N}{\hat{A}}_{B}^{T}-A_{N}A_{N}^{T}\hat{R},$$

(14) $${\hat{A}}_{B}=\left[\begin{array}{ccc} {\hat{v}}_{1} & {\hat{v}}_{2} & {\hat{v}}_{1}\times {\hat{v}}_{2} \end{array}\right].$$

Consequently, with the feedback, the error dynamics of $\sum_{1}$ are

(15) $$\sum_{1}:\left\{ \begin{array}{l} \dot{\hat{R}}=\hat{R}S\left(\omega_{m}-{\hat{b}}_{\omega }\right)+\theta K_{P}\hat{\Gamma }\\ {\dot{\hat{b}}}_{\omega }=\mathrm{Proj}\left(\left\|{\hat{b}}_{\omega }\right\|\leq l_{\hat{b}},-k_{v}\mathrm{vex}\left({\mathbb{P}}_{a}\left({\hat{R}}_{s}^{T}K_{P}\hat{\Gamma }\right)\right)\right) \end{array}\right..$$

Notice that, in Equation (9), J has been changed by Γ to facilitate subsequent derivation, because the second term of Γ is to ensure that $\left\|\tilde{\Gamma }\right\|$ is a linearity to $\left\|{\tilde{A}}_{B}\right\|$, where $\tilde{\Gamma }:=\Gamma -\hat{\Gamma }$ and ${\tilde{A}}_{B}:=A_{B}-{\hat{A}}_{B}$.

### 3.2. Subsystem $\sum_{2}$: Auxiliary Observer

**Figure 2:**
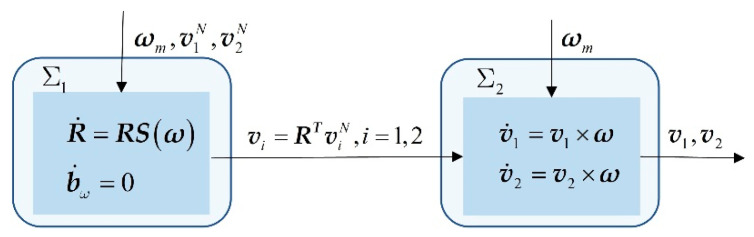
Structure of the nonlinear $\sum_{1}$ and linear $\sum_{2}$ interconnected system.

To weaken the injection of vector measurement noise in $\sum_{1}$, we determine that the vector measurements are not available as the input of $\sum_{1}$, as seen in Figure 2. Consequently, in this stage, we design an auxiliary observer whose role is to estimate the vector measurements for feeding the attitude observer. Since $v_{1}^{N}$ and $v_{2}^{N}$ are constants, from Equations (5) and (6), the derivative of the vector measurements is given by

(16) $$\left\{ \begin{array}{l} {\dot{v}}_{1}=v_{1}\times \omega \\ {\dot{v}}_{2}=v_{2}\times \omega \end{array}\right..$$

Combine the above equations with the measurement equation, Equation (6), and let i=1,2. Then, to estimate the states of the linear system in Equation (16), give the observer in a noiseless scenario:

(17) $$\sum_{2}:\left\{ \begin{array}{l} {\dot{\hat{v}}}_{1}={\hat{v}}_{1}\times \left(\omega_{m}-{\hat{b}}_{\omega }\right)+K_{1}\left(v_{1}-{\hat{v}}_{1}\right)\\ {\dot{\hat{v}}}_{2}={\hat{v}}_{2}\times \left(\omega_{m}-{\hat{b}}_{\omega }\right)+K_{2}\left(v_{2}-{\hat{v}}_{2}\right) \end{array}\right.,$$

where the gain matrices $K_{1}$ and $K_{2}$ are positive. Notice that the auxiliary observer is closely related to that introduced by Martin et al. [22]. The main difference between them is that the auxiliary observer uses the estimated gyro bias as an input rather than an estimated state, and the error system of $\sum_{2}$ is linear. Compared with the extended linear cascade subsystem in [15], our framework can be viewed as a simplified version because it only contains the additional items.

What is noteworthy is that the structure of the auxiliary observer $\sum_{2}$ makes it possible to use time-varying reference vectors in the inertial frame, since the knowledge of the constant vectors $v_{1}^{N}$ and $v_{2}^{N}$ is not employed.

### 3.3. Stability Analysis

To analyze the stability, define the estimation errors of $\sum_{2}$ as ${\tilde{v}}_{1}=v_{1}-{\hat{v}}_{1}$, ${\tilde{v}}_{2}=v_{2}-{\hat{v}}_{2}$. The error dynamics of system $\sum_{2}$ are

(18) $$\left\{ \begin{array}{l} {\dot{\tilde{v}}}_{1}=-\left(\omega_{m}-{\hat{b}}_{\omega }\right)\times {\tilde{v}}_{1}-K_{1}{\tilde{v}}_{1}+{\tilde{d}}_{1}\\ {\dot{\tilde{v}}}_{2}=-\left(\omega_{m}-{\hat{b}}_{\omega }\right)\times {\tilde{v}}_{2}-K_{2}{\tilde{v}}_{2}+{\tilde{d}}_{2} \end{array}\right.,$$

where $\tilde{d}={\left[{\tilde{d}}_{1},{\tilde{d}}_{2}\right]}^{T}={\left[-v_{1}\times {\tilde{b}}_{\omega },-v_{2}\times {\tilde{b}}_{\omega }\right]}^{T}$ is viewed as an input. Since $v_{1}$ and $v_{2}$ are uniformly bounded and $\left\|S\left(v_{1}\right)\right\|=\sqrt{2}\left\|v_{1}\right\|$, then $\left\|\tilde{d}\right\|\leq \sqrt{2}{\left({\left\|v_{1}\right\|}^{2}{\left\|{\tilde{b}}_{\omega }\right\|}^{2}+{\left\|v_{2}\right\|}^{2}{\left\|{\tilde{b}}_{\omega }\right\|}^{2}\right)}^{1/2}\leq L_{1}\left\|{\tilde{b}}_{\omega }\right\|$ for some $L_{1}>0$, which satisfies Assumption 3 in [15]. Let $\tilde{v}={\left[{\tilde{v}}_{1},{\tilde{v}}_{2}\right]}^{T}$ and transform the error dynamics in Equation (18) into a linear time-varying (LTV) error system:

(19) $$\dot{\tilde{v}}=\left(A-KC\right)\tilde{v}+B\tilde{d},$$

where $A=\left[\begin{array}{cc} -S\left(\omega_{m}-{\hat{b}}_{\omega }\right) & 0_{3\times 3}\\ 0_{3\times 3} & -S\left(\omega_{m}-{\hat{b}}_{\omega }\right) \end{array}\right]$, $B=\left[\begin{array}{cc} I_{3\times 3} & 0_{3\times 3}\\ 0_{3\times 3} & I_{3\times 3} \end{array}\right]$, $C=\left[\begin{array}{cc} I_{3\times 3} & 0_{3\times 3}\\ 0_{3\times 3} & I_{3\times 3} \end{array}\right]$, $K=\left[\begin{array}{cc} K_{1} & 0_{3\times 3}\\ 0_{3\times 3} & K_{2} \end{array}\right]$. To guarantee the stability of the observer error dynamics, K must be selected to make sure that the matrix A−KC is Hurwritz, as stated in Theorem 1 below.

According to the definition of $\hat{\Gamma }$ in the present paper, the error dynamics of $\sum_{1}$ are

(20) $$\left\{ \begin{array}{l} \dot{\tilde{R}}=RS\left(\omega_{m}-b_{\omega }\right)-\hat{R}S\left(\omega_{m}-{\hat{b}}_{\omega }\right)-\theta K_{P}\hat{\Gamma }\\ {\dot{\tilde{b}}}_{\omega }=-\mathrm{Proj}\left({\hat{b}}_{\omega },-k_{v}\mathrm{vex}\left({\mathbb{P}}_{a}\left({\hat{R}}_{s}^{T}K_{P}\hat{\Gamma }\right)\right)\right) \end{array}\right..$$

**Theorem** **1.** *Let $H_{K}\left(s\right)={\left(Is-A+KC\right)}^{-1}B$, and let θ be chosen to warrant stability. There exists a κ>0, such that if K is chosen to assure that A−KC is Hurwritz and ${\left\|H_{K}\left(s\right)\right\|}_{\infty }<\kappa$, then the origin of Equations (19) and (20) are GES. Moreover, a K that fulfills these conditions can be found all the time.*

**Proof.**  This proof acts similarly to [18]. First, (A,C) and (A,B) are easily verified to be observable and controllable, respectively. The pair (A,B,C) is left-invertible. According to the properties of the skew-symmetric matrix, we can infer that (A,B,C) is the minimum phase. As mentioned above, K should be chosen to fulfill the conditions of Theorem 1, and the same as for proof of Lemma 1, $\left\|{\hat{b}}_{\omega }\right\|$ must be in the definition domain of $\left\|{\hat{b}}_{\omega }\right\|\leq l_{\hat{b}}$. Next, choosing the same Lyapunov function candidate V as in [18], we get the equation of $\dot{V}$, which is

(21) $$\begin{array}{ll} \dot{V}\leq & -\beta_{2}\left({\left\|\tilde{R}\right\|}^{2}+{\left\|{\tilde{b}}_{\omega }\right\|}^{2}\right)+\mathrm{tr}\left({\tilde{R}}^{T}\theta K_{P}\tilde{\Gamma }\right)\\ & -\mu \mathrm{tr}\left(S\left(\mathrm{Proj}\left({\hat{b}}_{\omega },\tau \left(\Gamma \right)\right)-\mathrm{Proj}\left({\hat{b}}_{\omega },\tau \left(\hat{\Gamma }\right)\right)\right)R^{T}\tilde{R}\right)\\ & -\mu \mathrm{tr}\left(S\left({\tilde{b}}_{\omega }\right)R^{T}\theta K_{P}\tilde{\Gamma }\right)\\ & +\frac{2\theta \mu }{k_{v}}{\tilde{b}}_{\omega }^{T}\left(\mathrm{Proj}\left({\hat{b}}_{\omega },\tau \left(\Gamma \right)\right)-\mathrm{Proj}\left({\hat{b}}_{\omega },\tau \left(\hat{\Gamma }\right)\right)\right) \end{array}$$

where $\tau \left(\Gamma \right)=-k_{v}\mathrm{vex}\left({\mathbb{P}}_{a}\left({\hat{R}}_{s}^{T}K_{P}\Gamma \right)\right)$, and recall that $\tilde{\Gamma }=\Gamma -\hat{\Gamma }$. Write $\tilde{\Gamma }=A_{N}{\left(A_{B}-{\hat{A}}_{B}\right)}^{T}=A_{N}{\tilde{A}}_{B}{}^{T}$; it follows that $\left\|\tilde{\Gamma }\right\|\leq \left\|A_{N}\right\|\left\|{\tilde{A}}_{B}\right\|$, where $\left\|A_{N}\right\|\leq L_{A}$; see Property 1. Using the inequality ${\left\|v_{1}\times v_{2}\right\|}^{2}\leq 2\sqrt{2}\left({\left\|v_{1}\right\|}^{2}+{\left\|v_{2}\right\|}^{2}\right)$ derived from the inequality in [34], we can bound $\left\|{\tilde{A}}_{B}\right\|$ in the following way: Let ${\tilde{A}}_{B}=A_{B}-{\hat{A}}_{B}=[\begin{array}{ccc} v_{1}-{\hat{v}}_{1} & v_{2}-{\hat{v}}_{2} & v_{1}\times v_{2}-{\hat{v}}_{1}\times {\hat{v}}_{2} \end{array}]$, ${\tilde{v}}_{1}=v_{1}-{\hat{v}}_{1}$, and ${\tilde{v}}_{2}=v_{2}-{\hat{v}}_{2}$, then we have

(22) $$\begin{array}{ll} \left\|S\left(v_{1}\right)v_{2}-S\left({\hat{v}}_{1}\right){\hat{v}}_{2}\right\| & =\left\|S\left(v_{1}\right)v_{2}-S\left(v_{1}-{\tilde{v}}_{1}\right)\left(v_{2}-{\tilde{v}}_{2}\right)\right\|\\ & =\left\|S\left(v_{1}\right){\tilde{v}}_{2}+S\left({\tilde{v}}_{1}\right)v_{2}-S\left({\tilde{v}}_{1}\right)\left({\tilde{v}}_{2}\right)\right\|\\ & \leq \left\|S\left(v_{1}\right){\tilde{v}}_{2}\right\|+\left\|S\left({\tilde{v}}_{1}\right)v_{2}\right\|+\left\|S\left({\tilde{v}}_{1}\right)\left({\tilde{v}}_{2}\right)\right\|\\ & \leq \sqrt{2}\left(\left\|v_{1}\right\|\left\|{\tilde{v}}_{2}\right\|+\left\|{\tilde{v}}_{1}\right\|\left\|v_{2}\right\|+2{\left({\left\|{\tilde{v}}_{1}\right\|}^{2}+{\left\|{\tilde{v}}_{2}\right\|}^{2}\right)}^{1/2}\right)\\ & \leq \sqrt{2}\left(h_{1}\left\|{\tilde{v}}_{2}\right\|+h_{2}\left\|{\tilde{v}}_{1}\right\|+2{\left({\left\|{\tilde{v}}_{1}\right\|}^{2}+{\left\|{\tilde{v}}_{2}\right\|}^{2}\right)}^{1/2}\right)\\ & \leq h_{3}{\left({\left\|{\tilde{v}}_{1}\right\|}^{2}+{\left\|{\tilde{v}}_{2}\right\|}^{2}\right)}^{1/2} \end{array},$$

for some constant $h_{3}>0$, where $h_{1}$ and $h_{2}$ are bound on $v_{1}$ and $v_{2}$, respectively. Then the norm of ${\tilde{A}}_{B}$ becomes

(23) $$\begin{array}{ll} \left\|{\tilde{A}}_{B}\right\| & ={\left({\left\|{\tilde{v}}_{1}\right\|}^{2}+{\left\|{\tilde{v}}_{2}\right\|}^{2}+{\left\|S\left(v_{1}\right)v_{2}-S\left({\hat{v}}_{1}\right){\hat{v}}_{2}\right\|}^{2}\right)}^{1/2}\\ & \leq {\left({\left\|{\tilde{v}}_{1}\right\|}^{2}+{\left\|{\tilde{v}}_{2}\right\|}^{2}+h_{3}^{2}\left({\left\|{\tilde{v}}_{1}\right\|}^{2}+{\left\|{\tilde{v}}_{2}\right\|}^{2}\right)\right)}^{1/2}\\ & \leq \gamma_{1}{\left({\left\|{\tilde{v}}_{1}\right\|}^{2}+{\left\|{\tilde{v}}_{2}\right\|}^{2}\right)}^{1/2} \end{array}$$

for some $\gamma_{1}>0$. Given $\left\|\tilde{v}\right\|={\left({\left\|{\tilde{v}}_{1}\right\|}^{2}+{\left\|{\tilde{v}}_{2}\right\|}^{2}\right)}^{1/2}$, it is easy to get that $\left\|\tilde{\Gamma }\right\|\leq \left\|A_{N}\right\|\left\|{\tilde{A}}_{B}\right\|\leq L_{A}\gamma_{1}\left\|\tilde{v}\right\|$. Hence, $\left\|\theta K_{P}\tilde{\Gamma }\right\|\leq \theta L_{A}\gamma_{1}\left\|K_{P}\right\|\left\|\tilde{v}\right\|$. With the additional properties from Lemma 3 in [9], such that $\left\|\mathrm{Proj}\left({\hat{b}}_{\omega },\tau \right)\right\|\leq \tau$, $\left\|\mathrm{vex}\left({\mathbb{P}}_{a}\left(U\right)\right)\right\|\leq \left\|U\right\|/\sqrt{2}$, there exists a $\gamma_{2}>0$, such that $\left\|\tau \left(\Gamma \right)\right\|-\left\|\tau \left(\hat{\Gamma }\right)\right\|\leq \gamma_{2}\left\|\tilde{v}\right\|$. Likewise, there is a constant $\gamma_{3}>0$, such that $\left\|\mathrm{Proj}\left(\hat{b},\tau \left(\Gamma \right)\right)-\mathrm{Proj}\left(\hat{b},\tau \left(\hat{\Gamma }\right)\right)\right\|\leq \gamma_{3}\left\|\tilde{v}\right\|$. Then $\dot{V}$ becomes

(24) $$\begin{array}{ll} \dot{V} & \leq -\beta_{2}\left({\left\|\tilde{R}\right\|}^{2}+{\left\|{\tilde{b}}_{\omega }\right\|}^{2}\right)+\sqrt{3}\theta \left\|K_{P}\right\|\left|\gamma_{1}\right|\left\|\tilde{R}\right\|\left\|\tilde{v}\right\|\\ & \;\;\;+\sqrt{6}\mu \gamma_{3}\left\|\tilde{R}\right\|\left\|\tilde{v}\right\|+\sqrt{6}\mu \theta \left\|K_{P}\right\|\left|\gamma_{1}\right|\left\|{\tilde{b}}_{\omega }\right\|\left\|\tilde{v}\right\|+\frac{2\theta \mu \gamma_{3}}{k_{v}}\left\|{\tilde{b}}_{\omega }\right\|\left\|\tilde{v}\right\|\\ & \leq -\beta_{2}\xi^{2}+\gamma_{4}\xi \left\|\tilde{v}\right\| \end{array}$$

for some $\gamma_{4}>0$, $\beta_{2}>0$, where $\xi :={\left({\left\|\tilde{R}\right\|}^{2}+{\left\|{\tilde{b}}_{\omega }\right\|}^{2}\right)}^{1/2}$. □

Following the proof of Lemma 2 in [9], and using the same function $W={\tilde{v}}^{T}P\tilde{v}$ for some positive definite matrix P, one gets $\dot{W}\leq -{\left\|\tilde{v}\right\|}^{2}+\kappa^{2}{\left\|\tilde{d}\right\|}^{2}$. From the above analysis we can obtain $\left\|\tilde{d}\right\|\leq L_{1}\left\|{\tilde{b}}_{\omega }\right\|\leq L_{1}\left(\left\|\tilde{R}\right\|+\left\|{\tilde{b}}_{\omega }\right\|\right)$. Thus, $\dot{W}\leq -{\left\|\tilde{v}\right\|}^{2}+\kappa^{2}\gamma_{5}^{2}\xi^{2}$ for some $\gamma_{5}>0$. Considering the Lyapunov function Y=W+gV, we get $\dot{Y}<0$ for all adequately small κ. Therefore, this error system is GES.

### 3.4. Gain Selection

The task of this section is to choose the tunable gains. $K_{P}$, $k_{v}$ and θ of the attitude observer are chosen to conform to Lemma 1, and K of the auxiliary observer should be selected to secure the total observer’s stability. In practice, one can first choose arbitrary $K_{P}$, $k_{v}$, and then tune other gains to achieve stability, of which $K_{P}$ and K can be selected constants for improving the computational efficiency.

Additionally, according to the structure of Equation (18), we can find that it is straightforward to design time-varying gains for the auxiliary observer. One solution is to resolve the discrete time-varying Ricatti equation. This solution is similar to Kalman filter equations; the details are similar as in [25]. Furthermore, the utility of this method is that the noise and small interference items of the vector sensors can be under consideration. To make the derivations easier, we substitute $v_{i},\;i=1,2$ with $v_{i}+n_{i},\;i=1,2$ and replace $\omega_{m}$ with $\omega_{m}+\eta_{\omega }$ in Equation (17), like [28], to get the dynamics of the noisy auxiliary observer ${\sum^{\prime }}_{2}$

(25) $${\sum^{\prime }}_{2}:\left\{ \begin{array}{l} {\dot{\hat{v}}}_{1}={\hat{v}}_{1}\times \left(\omega_{m}+\eta_{\omega }-{\hat{b}}_{\omega }\right)+K_{1}\left(v_{1}+\eta_{1}-{\hat{v}}_{1}\right)\\ {\dot{\hat{v}}}_{2}={\hat{v}}_{2}\times \left(\omega_{m}+\eta_{\omega }-{\hat{b}}_{\omega }\right)+K_{2}\left(v_{2}+\eta_{2}-{\hat{v}}_{2}\right) \end{array}\right..$$

Then, from Equations (25) and (16), the estimation error dynamics of ${\sum^{\prime }}_{2}$ are

(26) $$\left\{ \begin{array}{l} {\dot{\tilde{v}}}_{1}={\dot{v}}_{1}-{\dot{\hat{v}}}_{1}=-S\left(\omega_{m}-{\hat{b}}_{\omega }\right){\tilde{v}}_{1}-K_{1}{\tilde{v}}_{1}+{\tilde{d}}_{1}+S\left({\hat{v}}_{1}\right)\eta_{\omega }-K_{1}\eta_{1}\\ {\dot{\tilde{v}}}_{2}={\dot{v}}_{2}-{\dot{\hat{v}}}_{2}=-S\left(\omega_{m}-{\hat{b}}_{\omega }\right){\tilde{v}}_{2}-K_{2}{\tilde{v}}_{2}+{\tilde{d}}_{2}+S\left({\hat{v}}_{2}\right)\eta_{\omega }-K_{2}\eta_{2} \end{array}\right..$$

Recall that $\tilde{d}={\left[{\tilde{d}}_{1},{\tilde{d}}_{2}\right]}^{T}={\left[-v_{1}\times {\tilde{b}}_{\omega },-v_{2}\times {\tilde{b}}_{\omega }\right]}^{T}$, $\tilde{v}={\left[{\tilde{v}}_{1},{\tilde{v}}_{2}\right]}^{T}$, given an input u, and $u=\tilde{d}$; thus, the LTV error system with noise terms is

(27) $$\dot{\tilde{v}}=\left(A-KC\right)\tilde{v}+Bu+B\eta_{u}-K\eta_{v},$$

where K and the (A,B,C) matrices are the same as before, $\eta_{u}$ is correlated with gyro noise, which gives $\eta_{u}={\left[S({\hat{v}}_{1})\eta_{\omega },S({\hat{v}}_{2})\eta_{\omega }\right]}^{T}$. $\eta_{v}$ is driven by vector measurement noise, having $\eta_{v}={\left[\eta_{1},\eta_{2}\right]}^{T}$. As stated in Assumption 1, the two vector measurements are noncorrelated, thus defining the process noise covariance matrix and measurement noise covariance matrix as $Q=\mathrm{blkdiag}\left(Q_{1},Q_{2}\right)$ and $R_{v}=\mathrm{blkdiag}\left(R_{v1},R_{v2}\right)$, respectively. Regarding Q, $Q_{1}=E[S({\hat{v}}_{1})\eta_{\omega }\eta_{\omega }^{T}S^{T}({\hat{v}}_{1})]$ and $Q_{2}=E[S({\hat{v}}_{2})\eta_{\omega }\eta_{\omega }^{T}S^{T}({\hat{v}}_{2})]$, which are obtained from the gyro measurement noise. Concerning R, the matrices $R_{v1}$ and $R_{v2}$ are from the vector measurement noise by $R_{v}{}_{1}=E[\eta_{1}\eta_{1}^{T}]$ and $R_{v2}=E[\eta_{2}\eta_{2}^{T}]$. For computing K, the following discrete Kalman filtering formulas can be used:

(28) $$P_{k|k-1}=F_{k}P_{k-1|k-1}F_{k}^{T}+Q_{k}$$

(29) $$K_{k}=P_{k|k-1}C_{k}^{T}{\left(C_{k}P_{k|k-1}C_{k}^{T}+R_{vk}\right)}^{-1}$$

(30) $$P_{k|k}=\left(I-K_{k}C_{k}\right)P_{k|k-1}$$

where ${\left(\cdot \right)}_{k}$ means the discrete matrix at time $t_{k}$. Given the discretization of the state matrices, $F_{k}=e^{A\Delta t}$ and $Q_{k}=\int_{t_{k}}^{t_{k+1}}e^{A\Delta t}BQB^{T}e^{A^{T}\Delta t}ds$, in which $\Delta t=t_{k+1}-t_{k}$ is the sampling interval. $P_{k|k-1}$ and $P_{k}$ denote the covariance of the estimated error $\tilde{v}$. In addition to producing time-varying gains, another advantage of this method is that it regards vector measurement noise at any time, and in some cases, smaller interference items can also be regarded as noise items.

## 4. Robustness to Noise

All analyses of the stability in Section 3.3 are performed under the assumption that there is a noise-free scenario. In fact, all sensors’ measurements cannot avoid noise interference. Our main work of this section is to analyze the observer’s robustness to the noise on sensor measurements. Toward this end, assume that all noise is bounded. We start with the noise of the rate gyro and vector sensors under consideration by substituting $\omega_{m}+\eta_{\omega }$ for $\omega_{m}$, $v_{i}+n_{i},\;i=1,2$ for $v_{i},\;i=1,2$. Hence, in the presence of bounded noise, the dynamics of the attitude observer Equation (8) are replaced with

(31) $${\sum^{\prime }}_{1}:{\left\{ \begin{array}{l} \dot{\hat{R}}=\hat{R}S\left(\omega_{m}+\eta_{\omega }-{\hat{b}}_{\omega }\right)+\theta K_{P}\bar{\Gamma }\\ {\dot{\hat{b}}}_{\omega }=\mathrm{Proj}\left(\left\|{\hat{b}}_{\omega }\right\|\leq l_{\hat{b}},-k_{v}\mathrm{vex}\left({\mathbb{P}}_{a}\left({\hat{R}}_{s}^{T}K_{P}\bar{\Gamma }\right)\right)\right) \end{array}\right.}_{{}_{}},$$

where $\bar{\Gamma }$ can be written as

(32) $$\bar{\Gamma }\left(v_{1}+\eta_{1},v_{1}^{N},v_{2}+\eta_{2},v_{2}^{N},\hat{R}\right):=A_{N}{\bar{A}}_{B}^{T}-A_{N}A_{N}^{T}\hat{R},$$

and ${\bar{A}}_{B}$ is denoted by

(33) $${\bar{A}}_{B}=\left[\begin{array}{ccc} v_{1}+\eta_{1} & v_{2}+\eta_{2} & \left(v_{1}+\eta_{1}\right) \end{array}\times \left(v_{2}+\eta_{2}\right)\right].$$

Then the error dynamics are

(34) $$\left\{ \begin{array}{l} \dot{\tilde{R}}=RS\left(\omega_{m}-b_{\omega }\right)-\hat{R}S\left(\omega_{m}+\eta_{\omega }-{\hat{b}}_{\omega }\right)-\theta K_{P}\bar{\Gamma }\\ {\dot{\tilde{b}}}_{\omega }=-\mathrm{Proj}\left({\hat{b}}_{\omega },-k_{v}\mathrm{vex}\left({\mathbb{P}}_{a}\left({\hat{R}}_{s}^{T}K_{P}\bar{\Gamma }\right)\right)\right) \end{array}\right..$$

Note that the noisy system dynamics and noisy error dynamics of the auxiliary observer are given by Equations (25) and (26).

**Assumption** **4.** *For all t≥0, the sensor measurement noise $\eta_{\omega }$ and $\eta_{i},\;i=1,2$ are all uniformly bounded.*

**Theorem** **2.** *Under the condition of Lemma 1, consider the attitude observer Equation (8), and let the input $u_{1}={\left[\eta_{\omega },\eta_{v}\right]}^{T}$ meet Assumption 4, then the attitude observer error dynamics Equation (34) are input-to-state stable.*

**Proof.**  See Appendix A. □

**Theorem** **3.** *Under the condition of Theorem 1, consider the auxiliary observer Equation (17), and let the input $u_{2}={\left[{\tilde{b}}_{\omega },\eta_{u},\eta_{v}\right]}^{T}$ meet Assumption 4, then the attitude observer error dynamics Equation (25) are input-to-state stable.*

**Proof.**  See Appendix B. □

**Remark** **1.** *The premise of Theorem 3 is that the gyro bias estimation errors are bounded under Theorem 2.*

## 5. Simulation Results and Discussion

In this section, via a simulated attitude estimation system, we evaluated the proposed nonlinear interconnected observer (named NLIO) by comparing it with the nonlinear observer (NLO) proposed by Grip et al. [18], the cascade observer (NLCO) discussed in [28], and the multiplicative extended Kalman filter (MEKF) [35]. With fixed gains and time-varying gains, we called the proposed observer NLIO-FG and NLIO-TV, respectively. Consider the attitude and heading reference system (AHRS) that consists of a triaxial rate gyro and two triaxial vector sensors: an accelerometer and a magnetometer. All sensors were low-cost and had a frequency of 100 Hz. The attitude trajectory in each simulation was driven by $\omega ={\left[0.1\sin\left(\left(\pi /12\right)t\right),-0.2\cos\left(\left(\pi /10\right)t\right),0.1\sin\left(\left(\pi /12\right)t\right)\right]}^{T}$, and lasted 500 s. Besides, the rate gyro bias was $b_{\omega }={\left[-0.017,-0.017,0.017\right]}^{T}\mathrm{rad}/s$. Assume that the gravitational and magnetic fields are available, denoted as $g^{N}={\left[0,\;0,\;g\right]}^{T}$ and $mag^{N}={\left[0.3128,\;0,\;0.4282\right]}^{T}G$, where $g=9.81m/s^{2}$. All sensor noise was assumed as Gaussian white noise, and the rate gyro noise followed $\eta_{\omega }∼N(0,\;{\left(10^{-3}\mathrm{rad}/s\right)}^{2})$. Here, two simulations were conducted to illustrate the performance of our proposed observer. We carried out 100 independent runs with each case and calculated the mean-absolute error (MAE) and root-mean-squared error (RMSE) of the Euler angles to assess the estimation accuracy. Note that, in this paper, we assumed that the acceleration of the rigid body is quite small and negligible compared with the gravity vector.

### 5.1. Simulation A: Comparsion of NLIO and NLO

In this section, we aim to compare the performance of the NLO and the NLIO, because the attitude observer frameworks of the NLO and the NLIO have many similarities, and the NLIO can be seen as a development of the NLO. Four cases are given as follows, with different initial attitude values and vector sensor noise.

In all four cases, we established two sets of tuning gains for attitude observers in the NLO and NLIO. These were $K_{P}=15I_{3\times 3}$(for NLO, NLIO-FG, and NLIO-TV) and $K_{P}=5I_{3\times 3}$(for NLO-a and NILO-TV-a). The scaling gain of the gyro bias observer was fixed to $k_{v}=0.2I_{3\times 3}$; the scaling parameter θ was fixed to θ=1. Meanwhile, the initial covariance matrix of the NLIO-TV was $P_{0|0}=\mathrm{blkdiag}\left(10^{-5}I_{3\times 3},\;5\times 10^{-7}I_{3\times 3}\right)$.

**Case** **1.** *The initial attitude was chosen randomly from a uniform distribution [−π, π], and the initial gyro bias was ${\left[0,\;0,\;0\right]}^{T}\mathrm{rad}/s$. The noise of the accelerometer and magnetometer was set to $\eta_{1}∼N(0,\;{\left(5\times 10^{-3}g\right)}^{2})$ and $\eta_{2}∼N(0,\;{\left(8\times 10^{-3}G\right)}^{2})$, respectively. In this simulation, there were random initial attitude values for all observers. The cascaded auxiliary observer gains of the NLIO-FG were chosen as $K_{1}=5.6I_{3\times 3}$, $K_{2}=3.3I_{3\times 3}$.*

**Figure 3:**
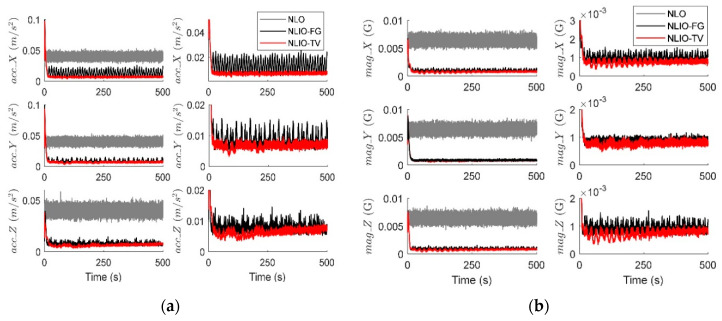
Averaged absolute vector measurement estimation errors over 100 simulations for Case 1. (**a**) Accelerometer measurement estimation errors evaluated by the auxiliary observers of the nonlinear observer (NLO) and nonlinear interconnected observer (NLIO). (**b**) Magnetometer measurement estimation errors evaluated by the auxiliary observers of the NLO and NLIOs. Since the NLO does not estimate the vector measurements, its vector measurement errors are identical to the difference between the sensor measurements and the truth values. The measurement errors’ formula is expressed as $\left|v_{m}-v_{truth}\right|$. For the NLIO, the vector measurement estimation error refers to the difference between the sensor measurement value and the truth value. Its formula is expressed as $\left|\hat{v}-v_{tuth}\right|$. To interpret the references to the color and line types in this figure, readers are invited to refer to the web version of this article.

**Figure 4:**
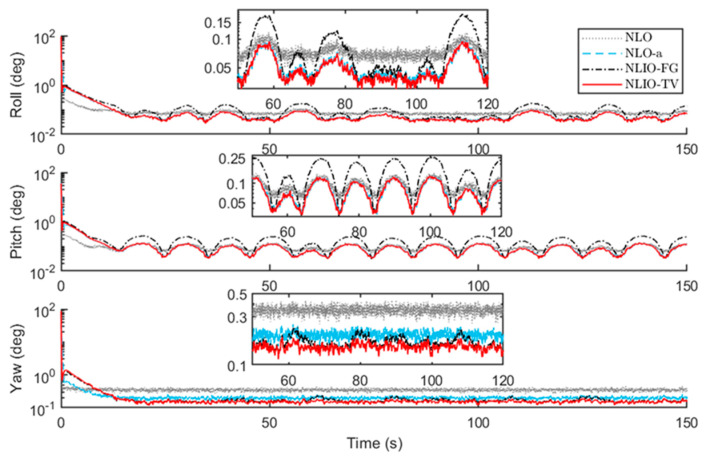
Averaged absolute attitude estimation error over 100 simulations for Case 1.

**Figure 5:**
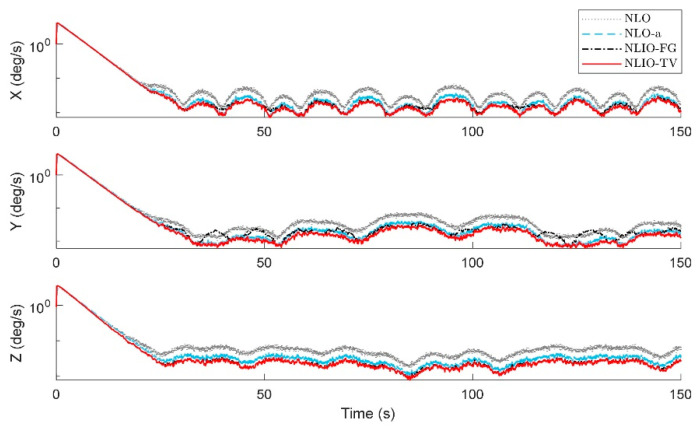
Averaged absolute gyro bias estimation errors over 100 simulations for Case 1.

From Figure 3, we can find that the auxiliary observers of the NLIOs can weaken the measurement noise and has good convergence, and the auxiliary observer with time-varying gains has better performance. Figure 4 and Figure 5 show the attitude and gyro bias estimation errors for each observer, respectively. These figures illustrate that the NLIO-TV slightly outperforms other observers by applying the time-varying gains to the auxiliary observer and using the estimated vector measurements as the attitude observer’s inputs. With the random initial attitude in [−π, π], we can see all observers can converge fast in the initial seconds of the transient phase, especially the NLIOs. Table 1 and Table 2 give the MAE values and RMSE values of the Euler angles, which represent the transient and steady-state performance of all observers. It is indicated that under the same attitude observer gains, the performance of the NLIO-FG is only better than the NLO in yaw. This means that a single auxiliary observer gain cannot meet the higher accuracy requirements of all the Euler angles.

**Case** **2.** *The initial attitude and gyro bias were ${\left[0,\;0,\;0\right]}^{T}\mathrm{rad}$ and ${\left[0,\;0,\;0\right]}^{T}\mathrm{rad}/s$; there was mixed-Gaussian noise of the accelerometer and magnetometer, which follow $\eta_{1}∼0.8N(0,\;{\left(5\times 10^{-3}g\right)}^{2})+0.2N(0,\;{\left(5\times 10^{-2}g\right)}^{2})$, $\eta_{2}∼0.8N(0,\;{\left(8\times 10^{-3}G\right)}^{2})+0.2N(0,\;{\left(8\times 10^{-2}G\right)}^{2})$.*

**Figure 6:**
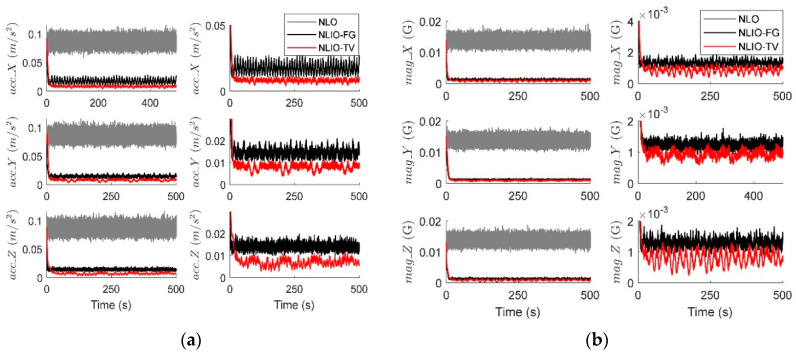
Averaged absolute vector measurement estimation errors over 100 simulations for Case 2. (**a**) Accelerometer measurement estimation errors evaluated by the auxiliary observers of the NLO and NLIOs. The first column: comparisons of NLO, NLIO-FG, and NLIO-TV. The second column: comparisons of NLIO-FG and NLIO-TV. (**b**) Magnetometer measurement estimation errors evaluated by the auxiliary observers of the NLO and NLIOs. The first column: comparisons of NLO, NLIO-FG, and NLIO-TV. The second column: comparisons of NLIO-FG and NLIO-TV.

**Figure 7:**
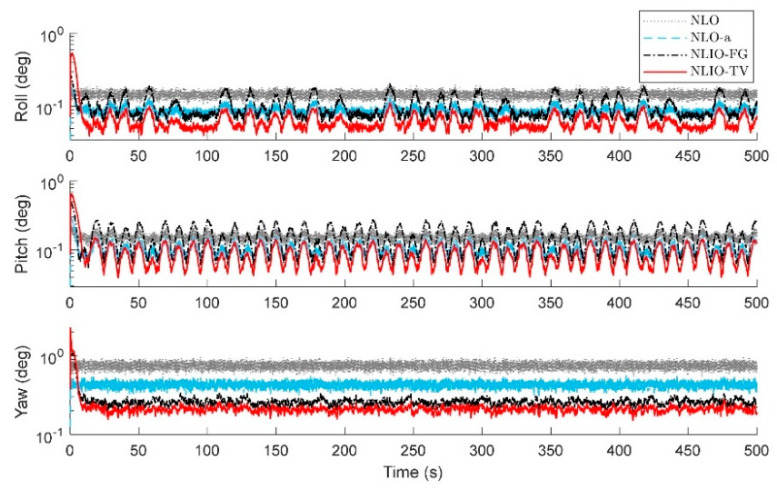
Averaged absolute attitude estimation error over 100 simulations for Case 2.

**Figure 8:**
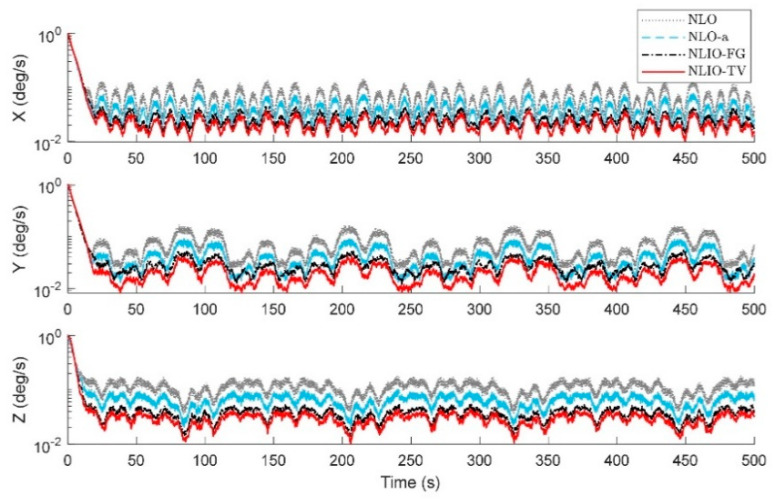
Averaged absolute gyro bias estimation errors over 100 simulations for Case 2.

In this case, we assumed the vector measurement noise follows a mixed Gaussian distribution. The proposed observer gains were $K_{1}=4.7I_{3\times 3}$ and $K_{2}=1.5I_{3\times 3}$. We can find in Figure 6 that the auxiliary observers of the NLIOs can minimize the measurement noise to a level similar to that made in Case 1. However, there is high-level noise on the vector sensors here compared to Case 1. Figure 7 and Figure 8 show that the NLIOs have better performance than the NLOs with identical $K_{P}$; obviously, with different tuning, the NLIO-TV is better than the NLO-a, which has an unaggressive $K_{P}$, performing significantly better than the NLO. The results of the NLIO-FG in pitch also show that a single auxiliary observer gain cannot satisfy all accuracy requirements. It can be seen from the steady-state MAEs and RMSEs in Table 3 and Table 4 that, compared to Case 1, the observers’ performance in Case 2 is degraded because of the mixed high-level noise. Yet, overall, the performance of the NLIO-TV and NLIO-FG is better than that of the NLO, especially the NLIO-TV.

**Figure 9:**
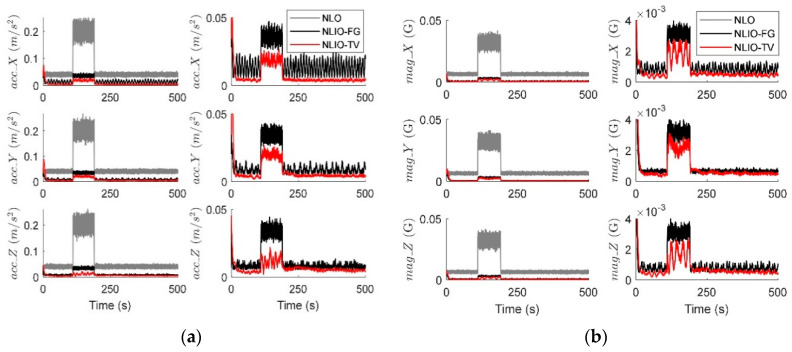
Averaged absolute vector measurement estimation errors over 100 simulations for Case 3. (**a**) Accelerometer measurement estimation errors evaluated by the auxiliary observers of the NLO and NLIOs. (**b**) Magnetometer measurement estimation errors evaluated by the auxiliary observers of the NLO and NLIOs.

**Figure 10:**
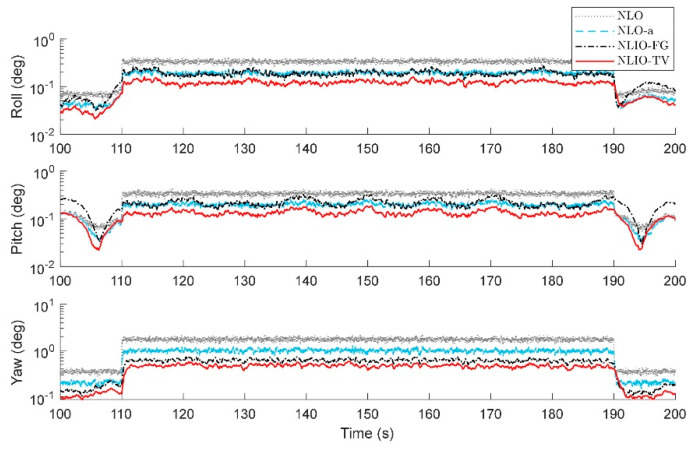
Averaged absolute attitude estimation error between 100 s and 200 s over 100 simulations for Case 3.

**Figure 11:**
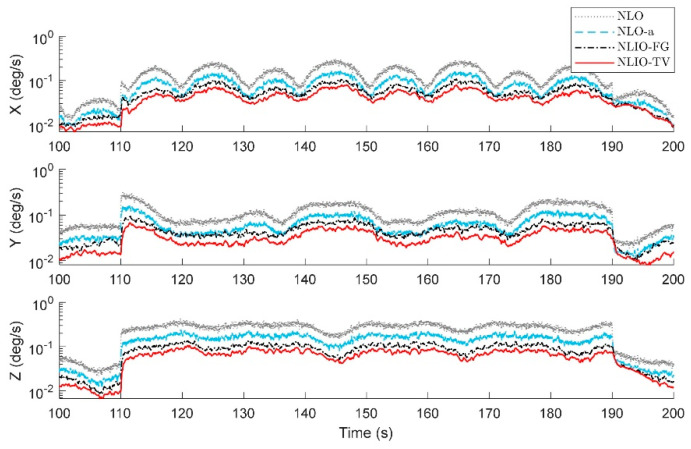
Averaged absolute gyro bias estimation errors between 100 s and 200 s over 100 simulations for Case 3.

**Case** **3.** *The initial values of the attitude and gyro bias were the same as in Case 2. The accelerometer noise and magnetometer noise were five times as the above cases, such that $\eta_{1}∼N(0,\;{\left(5\times 5\times 10^{-3}g\right)}^{2})$, $\eta_{2}∼N(0,\;{\left(5\times 8\times 10^{-3}G\right)}^{2})$ in 110s≤t≤190s. About this case, the proposed observer tuning gains were set to $K_{1}=5.5I_{3\times 3}$, $K_{2}=1.8I_{3\times 3}$. As seen in Figure 9, Figure 10 and Figure 11, between 110 s and 190 s, all observers are sensitive to the added noise, and the auxiliary observers of the NLIOs still have a strong attenuating effect on the added noise, especially that of the NLIO-TV. In general, the auxiliary observer with time-varying gains works better on reducing the impact of vector sensor noise in all cases. Moreover, the NLIO-TV still performs significantly better than the others.*

**Figure 12:**
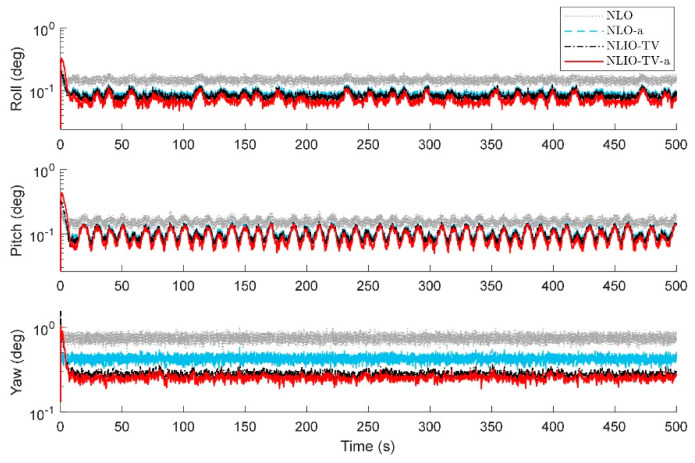
Averaged absolute attitude estimation error over 100 simulations.

**Figure 13:**
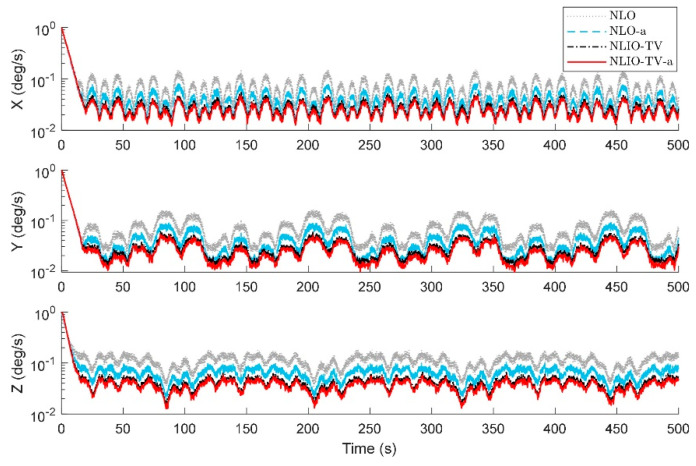
Averaged absolute gyro bias estimation errors over 100 simulations.

**Case** **4.** *In the above cases, we conclude that it is necessary to choose time-varying gains for the auxiliary observer. In order to compare the performance of the NLO and the NLIO-TV, we made the following comparison and still ran 100 times the same conditions of Case 2. The results in Figure 12 and Figure 13 show that the NLIOs have significant advantages in attitude estimation and gyro bias estimation than the NLOs. Furthermore, the MAEs in Table 5 prove this. Meanwhile, we can obtain that the NLIO has a lower sensitivity to $K_{P}$ than the NLO.*

### 5.2. Simulation B: Comparison of NLCO, MEKF, and NLIO-TV

In this section, we addressed a simulation to compare the performance of the NLIO-TV against the NLCO and the MEKF. The initial attitude was generated randomly from a uniform distribution [−π, π], and the initial gyro bias was ${\left[0,\;0,\;0\right]}^{T}\mathrm{rad}/s$. The noise of the accelerometer and magnetometer was set to $\eta_{1}∼0.8N(0,\;{\left(5\times 10^{-3}g\right)}^{2})+0.2N(0,\;{\left(5\times 10^{-2}g\right)}^{2})$, $\eta_{2}∼0.8N(0,\;{\left(8\times 10^{-3}G\right)}^{2})+0.2N(0,\;{\left(8\times 10^{-2}G\right)}^{2})$. In this case, the initial attitude values were randomly selected in a domain, and the vector measurement noise was mixed Gaussian noise. We chose the gyro bias parameters and the vector measurement parameters of the NLCO as $b_{1}=1.5$, $b_{1}=0.3$, and $m_{1}=m_{2}=0.02$. Fix the tuning gains for the attitude observers in the NLIO-TV as $K_{P}=1.5I_{3\times 3}$, $k_{v}=0.2I_{3\times 3}$. The initial covariance matrix of the NLIO-TV and the MEKF were $P_{NLIO\;0|0}=\mathrm{blkdiag}\left(10^{-5}I_{3\times 3},\;5\times 10^{-7}I_{3\times 3}\right)$ and $P_{MEKF\;0|0}=\mathrm{blkdiag}\left(10^{-4}I_{3\times 3},\;1\times 10^{-7}I_{3\times 3}\right)$, respectively.

**Figure 14:**
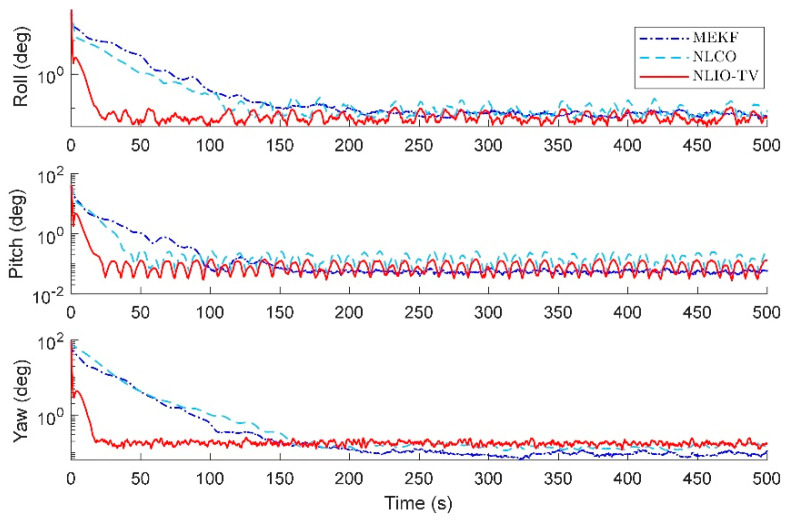
Averaged absolute attitude estimation error over 100 simulations.

**Figure 15:**
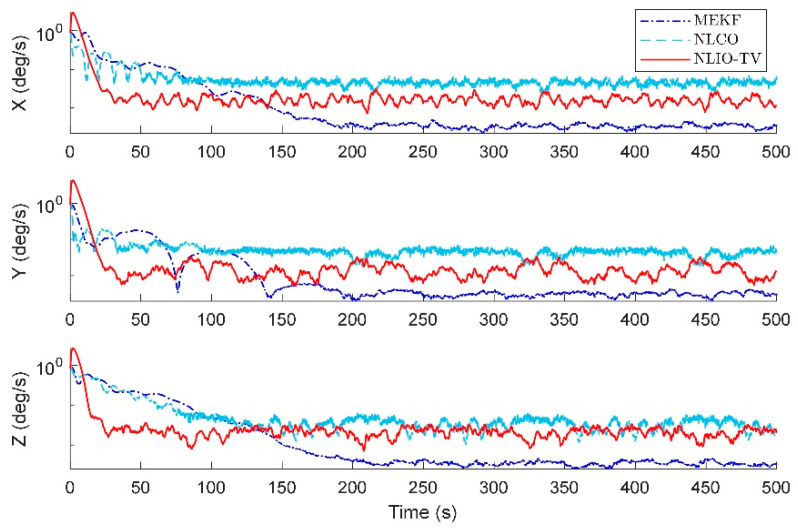
Averaged absolute gyro bias estimation errors over 100 simulations.

The results in Figure 14 and Figure 15 show that the NLIO-TV has notable advantages in attitude estimation and gyro bias estimation than the NLCO and MEKF in the transient phase. With large initial angle errors, the NLIO-TV converge more quickly than the NLCO and MEKF, which is because the MEKF only works well at small initial angle errors. In the steady-state phase, the MEKF performs better than the NLCO and NLIO-TV. Although MEKF performs well at the steady-state phase, it is a locally stable observer and requires strict initial conditions. Furthermore, the MAEs and RSMEs in Table 6 and Table 7 also record the excellent performance of NLIO-TV in the transient phase. Overall, the NLIO-TV is superior to the NLCO.

### 5.3. Discussion

Notice that the convergence speed of the attitude and gyro bias estimation errors can be controlled by tuning the attitude observer’s tunable parameters. As discussed in Section 3.4, cascaded observer tuning usually selects the proper values of $K_{P}$ and $k_{v}$ first, and then adjusts the linear system gains to get stability. Previous results show that the accuracy of the NLIO will not be greatly affected by $K_{P}$, which indicates that the auxiliary observer reduces part of the vector sensor noise before estimating the attitude and gyro bias. However, the selection of $K_{P}$ can influence the initial convergence rate of the NLIO.

Although the NLIO increases the computational burden, it can obtain more accurate estimates because of more adaptable and insensitive characteristics to the given $K_{P}$ and $k_{v}$. Furthermore, for the NLIO-FG, it is not easy to select a fixed gain for each channel, and constant adjustment is required to achieve excellent attitude estimation.

Therefore, the best choice is the NLIO-TV, which adds the gyro and vector measurement noise terms to the observer error model. By scaling Q and $R_{v}$, uncertain noise and small interference items can also be considered. Besides, when the vector measurement noise is small, the NLO can be used to save calculation time.

When vector measurements have high noise levels, the NLIO-TV has apparent advantages because of the following reasons:

- The auxiliary observer is designed by avoiding injecting more measurement noise, which is reflected in the estimated vector measurement used in the first term of the dynamic equation.
- The auxiliary observer weakens the measurement noise when estimating the vector measurements. Utilizing the estimated vector measurement for estimating the attitude and gyro bias can effectively improve accuracy and robustness.
- Noise terms are taken into account in its filtering part. The previous derivation shows that the NLIO-FG also has the first two advantages.

Lastly, according to Section 4, it is worth noting that as long as the noise terms or small interference terms are bounded, the estimation errors of the attitude, gyro bias, and vector measurement are bounded.

## 6. Conclusions

We have introduced an interconnected observer with global exponential stability for attitude and gyro bias estimation by designing its structure, analyzing its stability and robustness to noise, and evaluating its performance through simulation. To obtain better accuracy and robustness, we have further proposed a method to compute the time-varying gains of the auxiliary observer by adding noise terms to the error dynamics model. The simulation results showed that our approach with time-varying gains is conducive to the rapid convergence of attitude and gyro bias estimation and suppression of vector measurement noise compared with other nonlinear observers. Future work will concentrate on utilizing time-varying reference vectors in the proposed observer and extending the proposed observer in the GNSS/INS system, which can be simulated and validated using NaveGo [36].

## Figures and Tables

**Table 1:** MAEs of attitude for all the nonlinear observers in Case 1.

	Transient (0–200 s)			Steady-State (300–500 s)		
	Roll (deg)	Pitch (deg)	Yaw (deg)	Roll (deg)	Pitch (deg)	Yaw (deg)
NLO	0.6196	0.0985	0.3662	0.1149	0.0748	0.3495
NLO-a	1.0376	0.1233	0.2557	0.0920	0.0510	0.2015
NLIO-FG	0.6499	0.1200	0.2095	0.1661	0.0778	0.1689
NLIO-TV	0.6180	0.0875	0.2051	0.0930	0.0515	0.1661

**Table 2:** RMSEs of attitude for all the nonlinear observers in Case 1.

	Transient (0–200 s)			Steady-State (300–500 s)		
	Roll (deg)	Pitch (deg)	Yaw (deg)	Roll (deg)	Pitch (deg)	Yaw (deg)
NLO	1.0823	1.0171	0.1104	0.0936	0.4379	0.0933
NLO-a	1.7734	1.4817	0.1332	0.0635	0.2524	0.0772
NLIO-FG	1.0923	0.9448	0.1786	0.0961	0.2110	0.1450
NLIO-TV	1.0825	0.9442	0.1077	0.0641	0.2077	0.0776

**Table 3:** MAEs of attitude for all the observers in Case 2.

	Transient (0–200 s)			Steady-State (300–500 s)		
	Roll (deg)	Pitch (deg)	Yaw (deg)	Roll (deg)	Pitch (deg)	Yaw (deg)
NLO	0.1972	0.1478	0.7514	0.1968	0.1476	0.7498
NLO-a	0.1331	0.0904	0.4293	0.1295	0.0886	0.4280
NLIO-FG	0.1902	0.1037	0.2757	0.1836	0.0991	0.2566
NLIO-TV	0.1400	0.0726	0.2341	0.1061	0.0649	0.2253

**Table 4:** RMSEs of attitude for all the observers in Case 2.

	Transient (0–200 s)			Steady-State (300–500 s)		
	Roll (deg)	Pitch (deg)	Yaw (deg)	Roll (deg)	Pitch (deg)	Yaw (deg)
NLO	0.1853	0.9419	0.1576	0.1850	0.9400	0.1573
NLO-a	0.1133	0.5383	0.1069	0.1109	0.5364	0.1043
NLIO-FG	0.1289	0.3704	0.1585	0.1234	0.3213	0.1548
NLIO-TV	0.1079	0.3461	0.0990	0.0810	0.2820	0.0870

**Table 5:** MAEs of attitude for NLO and NLIO-TV with different parameters.

	Transient (0–200 s)			Steady-State (300–500 s)		
	Roll (deg)	Pitch (deg)	Yaw (deg)	Roll (deg)	Pitch (deg)	Yaw (deg)
NLO	0.1969	0.1480	0.7508	0.1968	0.1476	0.7523
NLO-a	0.1330	0.0904	0.4296	0.1296	0.0884	0.4302
NLIO-TV	0.1304	0.0870	0.2901	0.1252	0.0842	0.2818
NLIO-TV-a	0.1237	0.0761	0.2641	0.1112	0.0710	0.2537

**Table 6:** MAEs of attitude for all observers.

	Transient (0–200 s)			Steady-State (300–500 s)		
	Roll (deg)	Pitch (deg)	Yaw (deg)	Roll (deg)	Pitch (deg)	Yaw (deg)
MEKF	3.8376	2.9606	4.7822	0.0689	0.0681	0.0950
NLCO	4.6182	1.9867	6.9415	0.1667	0.0878	0.1477
NLIO-TV	2.1399	0.3371	0.5107	0.0964	0.0533	0.1777

**Table 7:** RMSEs of attitude for all observers.

	Transient (0–200 s)			Steady-State (300–500 s)		
	Roll (deg)	Pitch (deg)	Yaw (deg)	Roll (deg)	Pitch (deg)	Yaw (deg)
MEKF	6.5455	10.785	1.3573	0.0689	0.1178	0.0556
NLCO	7.0986	16.998	1.2130	0.1667	0.1798	0.1423
NLIO-TV	3.6250	3.3297	0.3448	0.0964	0.2216	0.0813

## Appendix A. Proof of Theorem 2

**Proof.** Proof. Under Assumption 4, consider the bounded input $u_{1}={\left[\eta_{\omega },\eta_{v}\right]}^{T}$, thus $\left\|\eta_{\omega }\right\|\leq \left\|u_{1}\right\|$, $\eta_{1}\leq \left\|u_{1}\right\|$ and $\eta_{2}\leq \left\|u_{1}\right\|$. From Equations (9) and (32), get that $\bar{\Gamma }-\Gamma =A_{N}{\bar{A}}_{B}^{T}-A_{N}A_{B}^{T}=A_{N}\left({\bar{A}}_{B}^{T}-A_{B}^{T}\right)$. Define $\varepsilon_{\Gamma }=\bar{\Gamma }-\Gamma$ and $\varepsilon :={\bar{A}}_{B}-A_{B}$, then it is possible to write $\varepsilon_{\Gamma }=A_{N}\varepsilon^{T}$, where $\varepsilon =[\eta_{1}\;\;\eta_{2}\;\;v_{1}\times \eta_{2}+\eta_{1}\times v_{2}+\eta_{1}\times \eta_{2}]$. Then the error dynamics of attitude observer Equation (34) can be written as

$$\left\{ \begin{array}{l} \dot{\tilde{R}}=\tilde{R}S\left(\omega_{m}-b_{\omega }\right)-\left(R-\tilde{R}\right)S\left({\tilde{b}}_{\omega }\right)-\left(R-\tilde{R}\right)S\left(\eta_{\omega }\right)-\theta K_{P}\Gamma -\theta K_{P}\varepsilon_{\Gamma }\\ {\dot{\tilde{b}}}_{\omega }=-\mathrm{proj}\left({\hat{b}}_{\omega },\tau \left(\Gamma \right)+\tau \left(\varepsilon_{\Gamma }\right)\right) \end{array}\right.$$

where $\tau \left(\varepsilon_{\Gamma }\right)=-k_{v}\mathrm{vex}\left({\mathbb{P}}_{a}\left({\hat{R}}_{s}^{T}K_{P}\varepsilon_{\Gamma }\right)\right)$. Using the Lyapunov-like function in [18],

$$V_{1}:=\frac{1}{2}{\left\|\tilde{R}\right\|}^{2}-\mu \mathrm{tr}\left(S\left({\tilde{b}}_{\omega }\right)R^{T}\tilde{R}\right)+\frac{\mu \theta }{k_{v}}{\tilde{b}}_{\omega }{\tilde{b}}_{\omega }^{T}$$

where 0<μ<1. Its derivative accompanying the above noisy error dynamics is

$$\begin{array}{ll} {\dot{V}}_{1} & =\mathrm{tr}\left({\tilde{R}}^{T}\dot{\tilde{R}}\right)-\mu \mathrm{tr}\left(S\left({\dot{\tilde{b}}}_{\omega }\right)R^{T}\tilde{R}\right)-\mu \mathrm{tr}\left(S\left({\tilde{b}}_{\omega }\right){\dot{R}}^{T}\tilde{R}\right)-\mu \mathrm{tr}\left(S\left({\tilde{b}}_{\omega }\right)R^{T}\dot{\tilde{R}}\right)+\frac{2\mu \theta }{k_{v}}{\tilde{b}}_{\omega }^{T}{\dot{\tilde{b}}}_{\omega }\\ & =\mathrm{tr}\left({\tilde{R}}^{T}\tilde{R}S\left(\omega_{m}-b_{\omega }\right)-{\tilde{R}}^{T}\left(R-\tilde{R}\right)S\left({\tilde{b}}_{\omega }\right)-{\tilde{R}}^{T}\left(R-\tilde{R}\right)S\left(\eta_{\omega }\right)-\theta {\tilde{R}}^{T}K_{P}\Gamma -\theta {\tilde{R}}^{T}K_{P}\varepsilon_{\Gamma }\right)\\ & +\mu \mathrm{tr}\left(S\left(\mathrm{proj}\left({\hat{b}}_{\omega },\tau \left(\Gamma \right)+\tau \left(\varepsilon_{\Gamma }\right)\right)\right)R^{T}\tilde{R}\right)-\mu \mathrm{tr}\left(S\left({\tilde{b}}_{\omega }\right)S^{T}\left(\omega_{m}-b_{\omega }\right)R^{T}\tilde{R}\right)\\ & -\mu \mathrm{tr}\left(S\left({\tilde{b}}_{\omega }\right)R^{T}\tilde{R}S\left(\omega_{m}-b_{\omega }+{\tilde{b}}_{\omega }\right)\right)+\mu \mathrm{tr}\left(S\left({\tilde{b}}_{\omega }\right)R^{T}\left(R-\tilde{R}\right)\left({\tilde{b}}_{\omega }\right)\right)+\mu \theta \mathrm{tr}\left(S\left({\tilde{b}}_{\omega }\right)R^{T}K_{P}\Gamma \right)\\ & +\mu \theta \mathrm{tr}\left(S\left({\tilde{b}}_{\omega }\right)R^{T}\left(R-\tilde{R}\right)S\left(\eta_{\omega }\right)\right)+\mu \theta \mathrm{tr}\left(S\left({\tilde{b}}_{\omega }\right)R^{T}K_{P}\varepsilon_{\Gamma }\right)-\frac{2\mu \theta }{k_{v}}{\tilde{b}}_{\omega }^{T}\left(\mathrm{proj}\left({\hat{b}}_{\omega },\tau \left(\Gamma \right)+\tau \left(\varepsilon_{\Gamma }\right)\right)\right) \end{array}$$

Combining the recalled equation $\Gamma =A_{N}A_{N}^{T}{\tilde{R}}^{T}$ with Property 1, we get

$$\begin{array}{ll} \mathrm{tr}\left({\tilde{R}}^{T}K_{P}\Gamma \right) & =\mathrm{tr}\left({\tilde{R}}^{T}K_{P}A_{N}A_{N}^{T}\tilde{R}\right)=\mathrm{tr}\left(K_{P}A_{N}A_{N}^{T}{\tilde{R}}^{T}\tilde{R}\right)\\ & \geq \lambda_{\min}\left(K_{P}\right)\lambda_{\min}\left(A_{N}A_{N}^{T}\right)\mathrm{tr}\left({\tilde{R}}^{T}\tilde{R}\right)\\ & =\lambda_{\min}\left(K_{P}\right)\lambda_{\min}\left(A_{N}A_{N}^{T}\right){\left\|\tilde{R}\right\|}^{2}\geq \beta_{1}\lambda_{\min}\left(K_{P}\right){\left\|\tilde{R}\right\|}^{2} \end{array}$$

where $\lambda_{\min}\left(K_{P}\right)$ denotes the minimum eigenvalue of $K_{P}$. In accordance with the definition of ε and $\varepsilon_{\Gamma }$, and the inequality ${\left\|v_{1}\times v_{2}\right\|}^{2}\leq 2\sqrt{2}\left({\left\|v_{1}\right\|}^{2}+{\left\|v_{2}\right\|}^{2}\right)$ appeared in preceding section, we can bound the above with

$$\begin{array}{ll} \left\|\varepsilon \right\|= & {\left({\left\|\eta_{1}\right\|}^{2}+{\left\|\eta_{2}\right\|}^{2}+{\left\|v_{1}\times \eta_{2}+\eta_{1}\times v_{2}+\eta_{1}\times \eta_{2}\right\|}^{2}\right)}^{1/2}\\ & \leq {\left(2{\left\|u_{1}\right\|}^{2}+2{\left\|v_{1}\right\|}^{2}{\left\|\eta_{2}\right\|}^{2}+2{\left\|v_{2}\right\|}^{2}{\left\|\eta_{1}\right\|}^{2}+2{\left\|\eta_{1}\times \eta_{2}\right\|}^{2}\right)}^{1/2}\\ & \leq {\left(2{\left\|u_{1}\right\|}^{2}+4L_{A}^{2}{\left\|u_{1}\right\|}^{2}+2\sqrt{2}\left({\left\|\eta_{1}\right\|}^{2}+{\left\|\eta_{2}\right\|}^{2}\right)\right)}^{1/2}\\ & \leq {\left(\left(2+4L_{A}^{2}\right){\left\|u_{1}\right\|}^{2}+4\sqrt{2}{\left\|u_{1}\right\|}^{2}\right)}^{1/2}={\left(2+4\sqrt{2}+4L_{A}^{2}\right)}^{1/2}\left\|u_{1}\right\| \end{array}$$

thus, $\left\|\varepsilon_{\Gamma }\right\|\leq \left\|A_{N}\right\|\left\|\varepsilon \right\|\leq L_{A}{\left(2+4\sqrt{2}+4L_{A}^{2}\right)}^{1/2}\left\|u_{1}\right\|\leq \beta_{3}\left\|u_{1}\right\|$, for some $\beta_{3}>0$. Then recall $\left|\mathrm{tr}\left(U\right)\right|\leq \sqrt{3}\left\|U\right\|$, thus $\mathrm{tr}\left({\tilde{R}}^{T}RS\left({\tilde{b}}_{\omega }\right)\right)\leq \sqrt{6}\left\|\tilde{R}\right\|\left\|{\tilde{b}}_{\omega }\right\|$, $\mathrm{tr}\left({\tilde{R}}^{T}RS\left(\eta_{\omega }\right)\right)\leq \sqrt{6}\left\|\tilde{R}\right\|\left\|u_{1}\right\|$, and

$$\begin{array}{ll} \left|\mathrm{tr}\left({\tilde{R}}^{T}K_{P}\varepsilon_{\Gamma }\right)\right| & \leq \sqrt{3}\left\|K_{P}{\tilde{R}}^{T}\varepsilon_{\Gamma }\right\|\leq \sqrt{3}\left\|K_{P}\right\|\left\|\tilde{R}\right\|\left\|\varepsilon_{\Gamma }\right\|\\ & \leq \sqrt{3}\beta_{3}\left\|K_{P}\right\|\left\|\tilde{R}\right\|\left\|u_{1}\right\|\leq \beta_{4}\left\|\tilde{R}\right\|\left\|u_{1}\right\| \end{array}$$

for some $\beta_{4}>0$. Then using some properties mentioned in Section 2, the first term of ${\dot{V}}_{1}$ is bounded by $-\theta \beta_{1}\lambda_{\min}\left(K_{P}\right){\left\|\tilde{R}\right\|}^{2}+\sqrt{6}\left\|\tilde{R}\right\|\left\|{\tilde{b}}_{\omega }\right\|+\sqrt{6}\left\|\tilde{R}\right\|\left\|u_{1}\right\|+\theta \beta_{4}\left\|\tilde{R}\right\|\left\|u_{1}\right\|$. Under Assumption 2 and 3, we give $\left\|\omega_{m}-b_{\omega }\right\|\leq L_{\omega }$, $\left\|{\tilde{b}}_{\omega }\right\|\leq L_{\tilde{b}}$, such that the third term is bounded by $2\sqrt{3}\mu L_{\omega }\left\|\tilde{R}\right\|\left\|{\tilde{b}}_{\omega }\right\|$. Similarity, the fourth term is bounded by $2\sqrt{3}\mu \left(L_{\omega }+L_{\tilde{b}}\right)\left\|\tilde{R}\right\|\left\|{\tilde{b}}_{\omega }\right\|$, and the bounds on the seventh and eighth terms are $2\sqrt{3}\mu \left\|{\tilde{b}}_{\omega }\right\|\left\|u_{1}\right\|+2\sqrt{3}\mu L_{b}\left\|\tilde{R}\right\|\left\|u_{1}\right\|$ and $\sqrt{6}\theta \mu \beta_{3}\left\|K_{P}\right\|\left\|{\tilde{b}}_{\omega }\right\|\left\|u_{1}\right\|$. According to the properties of matrix trace, we can bound the fifth term by $-2\mu {\left\|{\tilde{b}}_{\omega }\right\|}^{2}$. For the second term, recalling that $\left\|\mathrm{Proj}\left({\hat{b}}_{\omega },\tau \right)\right\|\leq \tau$, then we obtain $\left\|\mathrm{Proj}\left({\hat{b}}_{\omega },\tau \left(\Gamma \right)\right)\right\|\leq 3\sqrt{2}\left|k_{v}\right|\left\|K_{P}\right\|L_{A}^{2}\left\|\tilde{R}\right\|/2$, $\left\|\mathrm{Proj}\left({\hat{b}}_{\omega },\tau \left(\varepsilon_{\Gamma }\right)\right)\right\|\leq 3\sqrt{2}\left\|K_{P}\right\|L_{A}^{2}\beta_{3}\left\|u_{1}\right\|/2$, therefore, we have

$$\mu \mathrm{tr}\left(S\left(\mathrm{proj}\left({\hat{b}}_{\omega },\tau \left(\Gamma \right)+\tau \left(\varepsilon_{\Gamma }\right)\right)\right)R^{T}\tilde{R}\right)\leq 3\sqrt{3}\mu \left|k_{v}\right|\left\|K_{P}\right\|L_{A}^{2}{\left\|\tilde{R}\right\|}^{2}+3\sqrt{3}\mu \left\|K_{P}\right\|L_{A}^{2}\beta_{3}\left\|u_{1}\right\|\left\|\tilde{R}\right\|$$

Think of the sixth term and the last term together, and use the inequality in [18], giving

$$\begin{array}{l} \mu \theta \mathrm{tr}\left(S\left({\tilde{b}}_{\omega }\right)R^{T}K_{P}\Gamma \right)-\frac{2\mu \theta }{k_{v}}{\tilde{b}}_{\omega }^{T}\left(\mathrm{proj}\left({\hat{b}}_{\omega },\tau \left(\Gamma \right)+\tau \left(\varepsilon_{\Gamma }\right)\right)\right)\\ \leq \sqrt{6}\mu \theta \left\|K_{P}\right\|L_{\tilde{b}}L_{A}^{2}{\left\|\tilde{R}\right\|}^{2}-\mu \theta \mathrm{tr}\left(S\left({\tilde{b}}_{\omega }\right)R_{s}^{T}K_{P}\varepsilon_{\Gamma }\right)\\ \leq \sqrt{6}\mu \theta \left\|K_{P}\right\|L_{\tilde{b}}L_{A}^{2}{\left\|\tilde{R}\right\|}^{2}+\sqrt{6}\mu \theta \beta_{3}\left\|K_{P}\right\|L_{\tilde{b}}\left\|\tilde{R}\right\|\left\|u_{1}\right\| \end{array}$$

Combine all terms of $\dot{V}$, and then we have

$$\begin{array}{ll} {\dot{V}}_{1} & \leq -\theta \beta_{1}\lambda_{\min}\left(K_{P}\right){\left\|\tilde{R}\right\|}^{2}+\sqrt{6}\left\|\tilde{R}\right\|\left\|{\tilde{b}}_{\omega }\right\|+\sqrt{6}\left\|\tilde{R}\right\|\left\|u_{1}\right\|+\theta \beta_{4}\left\|\tilde{R}\right\|\left\|u_{1}\right\|\\ & +3\sqrt{3}\mu \left|k_{v}\right|\left\|K_{P}\right\|L_{A}^{2}{\left\|\tilde{R}\right\|}^{2}+3\sqrt{3}\mu \left\|K_{P}\right\|L_{A}^{2}\beta_{3}\left\|\tilde{R}\right\|\left\|u_{1}\right\|+2\sqrt{3}\mu L_{\omega }\left\|\tilde{R}\right\|\left\|{\tilde{b}}_{\omega }\right\|\\ & +2\sqrt{3}\mu \left(L_{\omega }+L_{\tilde{b}}\right)\left\|\tilde{R}\right\|\left\|{\tilde{b}}_{\omega }\right\|-2\mu {\left\|{\tilde{b}}_{\omega }\right\|}^{2}+2\sqrt{3}\mu \left\|{\tilde{b}}_{\omega }\right\|\left\|u_{1}\right\|+2\sqrt{3}\mu L_{b}\left\|\tilde{R}\right\|\left\|u_{1}\right\|\\ & +\sqrt{6}\theta \mu \beta_{3}\left\|K_{P}\right\|\left\|{\tilde{b}}_{\omega }\right\|\left\|u_{1}\right\|+\sqrt{6}\theta \mu \left\|K_{P}\right\|L_{\tilde{b}}L_{A}^{2}{\left\|\tilde{R}\right\|}^{2}+\sqrt{6}\theta \mu \beta_{3}\left\|K_{P}\right\|L_{\tilde{b}}\left\|\tilde{R}\right\|\left\|u_{1}\right\|\\ & \leq -\beta_{2}\left({\left\|\tilde{R}\right\|}^{2}+{\left\|{\tilde{b}}_{\omega }\right\|}^{2}\right)+\beta_{5}\left(\left\|\tilde{R}\right\|+\left\|{\tilde{b}}_{\omega }\right\|\right)\left\|u_{1}\right\|\\ & \leq -\beta_{2}\xi^{2}+\beta_{6}\xi \left\|u_{1}\right\| \end{array}$$

for some $\beta_{2}>0$, $\beta_{5}>0$ and $\beta_{6}>0$. Where $\xi ={\left({\left\|\tilde{R}\right\|}^{2}+{\left\|{\tilde{b}}_{\omega }\right\|}^{2}\right)}^{1/2}$, we can see that ξ represents the norm of estimation error ${\left[\tilde{R},\;{\tilde{b}}_{\omega }\right]}^{T}$. Since $\beta_{6}>0$, given 0<ℓ<1, rewrite the above inequality as

$${\dot{V}}_{1}\leq -\beta_{2}\left(1-\ell \right)\xi^{2}-\beta_{2}\ell \xi \left(\xi -\frac{\beta_{6}}{\beta_{2}\ell }\left\|u_{1}\right\|\right)$$

Hence, for all $\xi \geq \beta_{6}\left\|u_{1}\right\|/\beta_{2}\ell$, there exists $\dot{V}\leq$ $-\beta_{2}\left(1-\ell \right)\xi^{2}$. This proof is invoking Theorem 4.19 in [37]. That means the error dynamics of the attitude observer are input-to-state stable with the bounded input signals. □

## Appendix B. Proof of Theorem 3

**Proof.** Under Assumption 4, consider the bounded input $u_{2}={\left[{\tilde{b}}_{\omega },\eta_{u},\eta_{v}\right]}^{T}$ and the estimated error $\tilde{x}={\left[{\tilde{v}}_{1},\;{\tilde{v}}_{2}\right]}^{T}$, then define the Lyapunov-like function ${\dot{V}}_{2}={\left\|\tilde{x}\right\|}^{2}/2$. Recall that $\left\|\tilde{d}\right\|\leq L_{1}\left\|{\tilde{b}}_{\omega }\right\|$ and K is positive, it is easy to indicate that

$$\begin{array}{l} {\dot{V}}_{2}=\frac{1}{2}\left({\dot{\tilde{x}}}^{T}\tilde{x}+{\tilde{x}}^{T}\dot{\tilde{x}}\right)\\ =\frac{1}{2}{\tilde{x}}^{T}\left(K+K^{T}\right)\tilde{x}+{\tilde{x}}^{T}\tilde{d}-{\tilde{x}}^{T}K\eta_{v}+{\tilde{x}}^{T}\eta_{u}\\ \leq -\lambda_{\min}\left(K\right){\left\|\tilde{x}\right\|}^{2}+\sqrt{2}L_{1}\left\|\tilde{x}\right\|\left\|u_{2}\right\|-\lambda_{\min}\left(K\right)\left\|\tilde{x}\right\|\left\|u_{2}\right\|+\left\|\tilde{x}\right\|\left\|u_{2}\right\|\\ =-\lambda {\left\|\tilde{x}\right\|}^{2}+\alpha \left\|\tilde{x}\right\|\left\|u_{2}\right\| \end{array}$$

where $\lambda =\lambda_{\min}\left(K\right)$, $\alpha =\sqrt{2}L_{1}+1-\lambda$. Fix 0<χ<1, the above inequality can be rewritten as

$${\dot{V}}_{2}\leq -\lambda \left(1-\chi \right){\left\|\tilde{x}\right\|}^{2}-\lambda \chi \left\|\tilde{x}\right\|\left(\left\|\tilde{x}\right\|-\frac{\alpha }{\lambda \chi }\left\|u_{2}\right\|\right)$$

Thus, for all $\left\|\tilde{x}\right\|\geq \alpha \left\|u_{2}\right\|/\lambda \chi$, such that ${\dot{V}}_{2}\leq -\lambda \left(1-\chi \right){\left\|\tilde{x}\right\|}^{2}$. This proof is invoking Theorem 4.19 in [37]. □
